# Assessment of Anthelmintic Efficacy of Mebendazole in School Children in Six Countries Where Soil-Transmitted Helminths Are Endemic

**DOI:** 10.1371/journal.pntd.0003204

**Published:** 2014-10-09

**Authors:** Bruno Levecke, Antonio Montresor, Marco Albonico, Shaali M. Ame, Jerzy M. Behnke, Jeffrey M. Bethony, Calvine D. Noumedem, Dirk Engels, Bertrand Guillard, Andrew C. Kotze, Alejandro J. Krolewiecki, James S. McCarthy, Zeleke Mekonnen, Maria V. Periago, Hem Sopheak, Louis-Albert Tchuem-Tchuenté, Tran Thanh Duong, Nguyen Thu Huong, Ahmed Zeynudin, Jozef Vercruysse

**Affiliations:** 1 Department of Virology, Parasitology and Immunology, Ghent University, Merelbeke, Belgium; 2 Department of Control of Neglected Tropical Diseases, World Health Organization, Geneva, Switzerland; 3 Fondazione Ivo de Carneri, Milan, Italy; 4 Public Health Laboratory-Ivo de Carneri, Chake Chake, United Republic of Tanzania; 5 School of Life Sciences, University of Nottingham, Nottingham, United Kingdom; 6 Microbiology, Immunology, and Tropical Medicine, George Washington University Medical Center, Washington, D.C., United States of America; 7 Centre for Schistosomiasis and Parasitology, University of Yaoundé I, Yaoundé, Cameroon; 8 Clinical Laboratory, Pasteur Institute, Phnom Penh, Cambodia; 9 Division of Animal, Food and Health Sciences, Commonwealth Scientific and Industrial Research Organisation, St. Lucia, Australia; 10 Instituto de Investigaciones en Enfermedades Tropicales, Universidad Nacional de Salta/CONICET, Oran, Argentina; 11 Queensland Institute for Medical Research, University of Queensland, Brisbane, Australia; 12 Department of Medical Laboratory Sciences and Pathology, Jimma University, Jimma, Ethiopia; 13 Instituto René Rachou, Fundação Oswaldo Cruz, Belo Horizonte, Brazil; 14 Department of Parasitology, National Institute of Malariology, Parasitology and Entomology, Ha Noi, Vietnam; World Health Organization, Switzerland

## Abstract

**Background:**

Robust reference values for fecal egg count reduction (FECR) rates of the most widely used anthelmintic drugs in preventive chemotherapy (PC) programs for controlling soil-transmitted helminths (STHs; *Ascaris lumbricoides*, *Trichuris trichiura*, and hookworm) are still lacking. However, they are urgently needed to ensure detection of reduced efficacies that are predicted to occur due to growing drug pressure. Here, using a standardized methodology, we assessed the FECR rate of a single oral dose of mebendazole (MEB; 500 mg) against STHs in six trials in school children in different locations around the world. Our results are compared with those previously obtained for similarly conducted trials of a single oral dose of albendazole (ALB; 400 mg).

**Methodology:**

The efficacy of MEB, as assessed by FECR, was determined in six trials involving 5,830 school children in Brazil, Cambodia, Cameroon, Ethiopia, United Republic of Tanzania, and Vietnam. The efficacy of MEB was compared to that of ALB as previously assessed in 8,841 school children in India and all the above-mentioned study sites, using identical methodologies.

**Principal Findings:**

The estimated FECR rate [95% confidence interval] of MEB was highest for *A. lumbricoides* (97.6% [95.8; 99.5]), followed by hookworm (79.6% [71.0; 88.3]). For *T. trichiura*, the estimated FECR rate was 63.1% [51.6; 74.6]. Compared to MEB, ALB was significantly more efficacious against hookworm (96.2% [91.1; 100], *p*<0.001) and only marginally, although significantly, better against *A. lumbricoides* infections (99.9% [99.0; 100], *p* = 0.012), but equally efficacious for *T. trichiura* infections (64.5% [44.4; 84.7], *p* = 0.906).

**Conclusions/Significance:**

A minimum FECR rate of 95% for *A. lumbricoides*, 70% for hookworm, and 50% for *T. trichiura* is expected in MEB-dependent PC programs. Lower FECR results may indicate the development of potential drug resistance.

## Author Summary

Soil-transmitted helminths (STHs; roundworms, whipworms, and hookworms) infect millions of children in sub-tropical and tropical countries, resulting in malnutrition, growth stunting, intellectual retardation, and cognitive deficits. To fight against STH, large-scale deworming programs are implemented in which anthelmintic drugs (either albendazole (ALB) or mebendazole (MEB)) are administered. Currently, these large-scale programs are intensifying, highlighting the need to closely monitor the efficacy of anthelmintic drugs to detect changes in drug efficacy that may arise through the evolution of anthelmintic drug resistance in the parasites. We have previously defined the minimum expected efficacy of ALB based on the fecal egg count reduction (FECR) rate, but these reference values are lacking for MEB. Therefore, we therefore evaluated the FECR rate of MEB against STHs in six STH endemic countries. In addition, we compared the results of the FECR rate for MEB with those we obtained previously for ALB. The results confirm that MEB treatment was highly efficacious against roundworms, and to a lesser extend against hookworms, but not against whipworms. Compared to ALB, MEB is less efficacious against hookworm, but equally efficacious against roundworms and whipworms. Based on this study we propose the minimum expected FECR rate for MEB-dependent large-scale deworming programs.

## Introduction

The soil-transmitted helminths (STHs, *Ascaris lumbricoides*, *Trichuris trichiura*, and the two hookworm species, *Necator americanus* and *Ancylostoma duodenale*) are responsible for the highest burden among all neglected tropical diseases (NTDs) [1]. Recent global estimates indicate that in 2010 more than 1.4 billion people were infected with at least one of the four STH species, resulting in a global burden of approximately 5.2 million disability-adjusted life years (DALYs) (20% of the total number of DALYs attributable to NTDs) [2]. Mass drug administration (MDA) programmes in which a single oral dose of albendazole (ALB) or mebendazole (MEB) - the drugs of choice for STHs - are periodically administered to pre-school and school aged children, are the main strategy for controlling the morbidity caused by STH [3], [4], and these programmes have recently received increased political and scientific attention [5], [6].

While the laudable long-term aim is to eliminate soil-transmitted helminthiasis as public problem by 2020 [7], the pledges of drug donations on this scale re-enforce the necessity for thoroughly designed monitoring systems that allow detection of any changes in anthelmintic drug efficacy that may arise through the evolution of anthelmintic drug resistance in these parasites. Both ALB and MEB belong to the same pharmaceutical group (benzimidazole drugs, BZ) sharing the same mode of action (the inhibition of the polymerisation of microtubules). Thus, development of resistance against any BZ drug would most likely by accompanied by poor anthelmintic drug efficacy of the other BZ drugs. It is pertinent also that there is a paucity of anthelmintic drugs licensed for the treatment of STH infections in humans and available commercially, and hence should anthelmintic resistance against BZ drugs eventually emerge and spread, chemotherapy based control of STHs will be even more limited than at present with few acceptable alternative options [8].

Currently, assessment of the reduction in fecal egg counts following drug administration (fecal egg count reduction (FECR) *syn.* egg reduction rate) is the recommended method for monitoring the efficacy of anthelmintic drugs against STHs [9]. In contrast to other available assays, it allows for the assessment of drug efficacy against all three STHs (*vs. in vitro* assays and molecular assays) with a minimum of laboratory equipment (*vs.* molecular assays) [10]–[12]. However, the interpretation of the results from the FECR tests remains difficult, since reliable reference efficacy values for BZ drugs (which can act as standard reference points for comparison with new data) for each of the STH species are still lacking. In a systematic review and meta-analysis of published efficacy trials targeting STH infections, Keiser and Utzinger (2008) [13] highlighted that there is a lack of high-quality trials to determine these reference values. The available efficacy data have been obtained through a variety of widely differing study protocols, including protocols that used different diagnostic methods, different durations in follow-up periods, differing origin of the drugs (i.e., different manufactures and therefore different quality), and statistical analyses, all of which impede a robust meta-analysis of drug efficacy based on FECR [13]. As a response to these earlier limitations, our consortium has recently reported on the efficacy of a single oral dose of ALB (400 mg) against STH in seven trials across sub-Saharan Africa, Asia, and Latin America based on a protocol standardized in respect to the origin of the drug (Zentel, GlaxoSmithKline, batch N° L298), the follow-up period (14 to 30 days), the egg counting method (McMaster egg counting method), the statistical analysis, and the interpretation of the data (group based FECR using arithmetic means) [14]. This study suggested that a FECR of 95% for *A. lumbricoides* and 90% for hookworm should be the expected minimum in all future drug efficacy studies, and that FECR rates below these levels following a single dose of ALB, should be viewed as danger signs of potential development of drug resistance. For *T. trichiura*, reference FECR values could only be provided for specified mean fecal egg count (FEC) values at pre-intervention, as the drug efficacy measured by FECR decreased as a function of increasing mean FEC at the pre-intervention survey. For *T. trichiura*, we therefore expect FECRs of at least 90%, 70%, and 50%, in populations where the mean FECs are below 275 eggs per 1 g of stool (EPG), 550 EPG, and 800 EPG, respectively, and even lower in settings where baseline infection intensities are higher [15]. Data derived from such standardized multi-center efficacy trials that establish reliable reference FECR values and assess the impact of infection intensity are still currently lacking for MEB. Therefore, in the present study we assessed the efficacy measured by means of FECR of a single oral dose of MEB (500 mg) against STHs in six trials in sub-Saharan Africa, Asia, and Latin America using a protocol that we previously standardized in assessing the drug efficacy of a single dose of ALB, and compared the drug efficacy of both BZ drugs against each of the STHs.

## Methods

### Ethics statement

The overall protocol of both the ALB and MEB trials was approved by the Ethics Committee of the Faculty of Medicine, Ghent University (reference nos. B67020084254 and 2011/374) and was followed by local ethical approval for each trial site. For Brazil, approval was obtained from the Institutional Review Board from Centro de Pesquisas René Rachou (no. 21/2008), for Cambodia from the National Ethics Committee for Health Research (no. 185 NECHR), for Cameroon from the National Ethics Committee (nos. 072/CNE/DNM08, 147/CNE/DNM/11), for India from the Institutional Review Board of the Christian Medical College (Vellore) (no. 6541; participated in the ALB study only), for Ethiopia from the Ethical Review Board of Jimma University (Jimma) (no. RPGE/09/2011), for United Republic of Tanzania from the Zanzibar Health Research Council (nos. 20, ZAMREC/0003/JUNE/2012), and for Vietnam by Ethical Committee of National Institute of Malariology, Parasitology and Entomology (Ha Noi) and the Ministry of Health (no. 752/QD-VSR). The parents of all subjects included in the studies signed an informed consent form. In Brazil and Ethiopia an informed consent form was obtained from children aged 10 or 11 years and above. In Cambodia and Ethiopia, a verbal assent was obtained from all children, and this procedure was approved by the respective ethics boards. Our studies assessing ALB and MEB are registered under ClinicalTrials.gov, identifiers nos. NCT01087099 and NCT01379326, respectively (CONSORT Checklist S1).

### Trial Sites

The MEB multi-center study reported here was carried out in six countries located in sub-Saharan Africa (Cameroon, Ethiopia, and United Republic of Tanzania (Zanzibar)), Asia (Cambodia and Vietnam), and Latin America (Brazil). However, it is important to note that, while we refer to individual countries to identify results from particular trials, names of countries are used only to distinguish between six separate trials that were conducted in six geographically distinct regions of the world. These six STH-endemic countries were selected because of the presence of investigator groups/institutions with extensive experience in the diagnosis, and control of STH. These same six investigator groups were also involved in the earlier evaluation of the efficacy of a single-oral dose 400 mg ALB against STH in children, based on an identical standardized protocol [14].

### Trial Design and Field Work

Since study designs can have a significant effect on the subsequent calculation of efficacy and to ensure that our values for ALB and MEB were not confounded by study design, the protocol described by Vercruysse and colleagues for assessment of ALB was also used here to evaluate the drug efficacy of MEB [14]. In short, schools were selected based on previous STH surveys. Within schools, children were recruited on a voluntary basis. School children aged 4–18 years at each of the different trial sites were asked to provide a stool sample during a pre-intervention survey. For the initial sampling the aim was to enroll at least 250 STH-infected children for at least one species. This sample size was selected based on statistical analysis of study power, using random simulations of correlated over-dispersed FEC data reflecting the variance-covariance structure in a selection of real FEC data sets. This analysis suggested that a sample size of up to 200 individuals (alpha = 0.05, power = 80%) was required to detect a 10% point drop from a null efficacy of 80% (mean FECR individual) over a wide range of infection scenarios. Standard power analyses for proportions also indicated that the detection of a 10% point drop from a null cure rate required sample sizes up to 200 (the largest samples being required to detect departures from null efficacies of around 50%). Given an anticipated non-compliance rate of 25%, a total of at least 250 individuals was therefore considered necessary at each study site. A single oral dose of 500 mg MEB (Vermox) from the same manufacturer (Janssen-Cilag, Latina, Italy, batch no: BCL2F00) was administered to the subjects at all study sites. Seven to 15 days after the pre-intervention survey (Brazil: 7–14 days; Cambodia: 11–15 days; Cameroon: 9–11 days; Ethiopia: 14 days; United Republic of Tanzania: 14 days; Vietnam: 11–12 days), stool samples were again collected from the subjects. Subjects who were unable to provide a stool sample at follow-up, or who were experiencing a severe intercurrent medical condition or had diarrhea at the time of the first sampling, were excluded from the study (Study Protocol S1).

### Laboratory Procedures

All stool samples were individually processed by the McMaster egg counting method. McMaster is a flotation technique that is commonly used in veterinary parasitology both to assess intensity of gastro-intestinal parasite infections and to evaluate drug efficacy against these parasites. For the diagnosis and enumeration of STHs in public health, it has been found to be user-friendly (*vs*. FLOTAC [16]) robust (*vs.* Kato-Katz thick smear [17]) and accurate for enumeration of STHs, but less sensitive when intensity of infection is low (*vs.* Kato-Katz and FLOTAC [16], [17])) Complementary data indicate that FECR estimates obtained by the McMaster are comparable to those using the Kato-Katz thick smear [18], [19].

The standard operating procedure to perform a McMaster on human stools is described in more detail elsewhere [15]. Briefly, 2 g of stool were suspended in 30 ml of saturated salt (NaCl) solution at room temperature (density: 1.2). The fecal suspension was poured three times through a tea sieve to remove large debris. After thorough mixing 10 times, 0.5 ml aliquots were added to each side of a McMaster slide chamber. Both chambers were examined under a light microscope using 100x magnification and the FEC, expressed as EPG for each helminth species, was obtained by multiplying the total number of eggs counted under the microscope by a factor 50. A detailed tutorial can be found on http://www.youtube.com/watch?v=UZ8tzswA3tc.

### Statistical Analysis

The efficacy of a single dose of MEB (500 mg) against each of the three STH species (the two hookworm species were treated as one species since the eggs of *A. duodenale* and *N. americanus* cannot be distinguished by conventional microscopy), as measured by FECR, was calculated for the different trials. We have not summarized the efficacy of MEB by means of cure rate (CR; the proportion of the subjects who are not excreting eggs after drug administration). This is because an intervention may fail to cure STH infections (CR = 0%), but may result in a FECR of 99%, which is satisfactory. Second, it has been shown that estimates of CR are highly affected by both sampling and diagnostic effort, estimates being overestimated when the sampling and diagnostic effort is minimized. This was in sharp contrast to FECR estimates, which remained unchanged regardless of both sampling and diagnostic effort [20].

To-date, a wide range of formulae has been used to calculate FECR, each differing in terms of the statistical unit (individual *vs.* group) and how the mean FEC is calculated (arithmetic *vs.* geometric). However, recent studies suggest that the group-based formula using the arithmetic mean, as described below, is a suitable metric for evaluating drug efficacy. Compared to the other formulae, it represents a robust indicator (*vs*. individual-based formula [14]) that provides accurate estimates of drug efficacy (*vs*. group-based formula using geometric mean) [14], [21]. Moreover, it is important to note that there is no common formula for calculating the variance for each of these FECR formulae. The formula used here to calculate variance for the group-based FECR using the arithmetic mean is described below and is based on the Delta method [22].
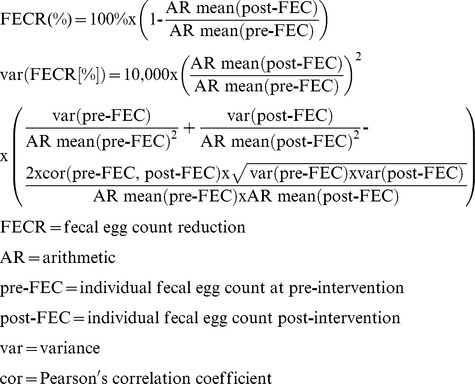



We first analyzed the trial outcomes for each of the three STHs separately using FECR data at the trial level, and its corresponding variance and sample size, and secondly we compared data from both the current trials with MEB and our earlier trials with ALB, employing meta-analytical approaches. In addition, the impact of infection intensity at the pre-intervention survey on the FECR rate of both BZ drugs was evaluated. To assess the FECR rate of MEB, generalized mixed effect models were fitted for each of the three STH species in turn, with the FECR rate at the trial level as the dependent variable, and the mean FEC at the pre-intervention survey as a covariate. To compare the FECR rates between ALB and MEB, generalized mixed effect models were fitted for each of the three STH species with FECR at the trial level as the dependent variable, and BZ drug (two levels: ALB and MEB) and mean FEC at the pre-intervention survey as covariates, and the interaction between these covariates. For both analyses, we only included trials for which at least 50 subjects who had been treated and provided stools at both pre- and post-intervention surveys were available. The different MEB and ALB trials included in the analyses are listed in Table 1 and Table 2, respectively. These tables also describe for each trial the sample size, mean age, sex ratio (number of female subjects/number of male subjects), mean FEC, and the level of infection intensity at the pre-intervention survey for each of the three STH species separately. The levels of infection intensity correspond to the low, moderate, and high intensity of infection ranges, as described by WHO [23]. For *A. lumbricoides* these were 1–4,999 EPG, 5,000–49,999 EPG, and ≥50,000 EPG; for *T. trichiura* these levels were 1–999 EPG, 1,000–9,999 EPG, and ≥10,000 EPG; and for hookworm these were 1–1,999 EPG, 2,000–3,999 EPG, and ≥4,000 EPG, respectively. The ALB trials have been described previously by Vercruysse et al. (2011) [14] and Mekonnen et al. (2013) [24], targeting *A. lumbricoides* (five trials), *T. trichiura* (five trials), and hookworm (7 trials). Each of these trials report FECR rates of a single dose ALB (400 mg), all were based on the aforementioned trial design, and with the exception of one trial (India) the same laboratories which assessed the FECR rates in current study were also involved in these ALB trials. The meta-analysis was carried out using the ‘metafor’ package of the statistical software R [25]. The level of significance was set at *p*<0.05.

**Table 1:** Number of infected subjects and characteristics of the study across six mebendazole trials.

	N	Sex ratio (Female:male)	Mean age (years)	Mean FEC (EPG)	Infection intensity level (%)			
					Low	Moderate		High
***A. lumbricoides***								
Brazil	133	1.18	9.3	8,078	55.6	43.6	0.8	
Cameroon	132	1.16	10.0	6,006	62.9	36.4	0.8	
Ethiopia	279	0.89	11.5	6,497	61.6	37.6	0.7	
Tanzania	378	1.20	10.7	6,761	61.9	37.3	0.8	
Vietnam	287	0.88	8.4	20,857	40.1	48.8	11.1	
***T. trichiura***								
Cameroon	175	0.82	9.9	420	89.1	10.9	0.0	
Ethiopia	326	1.16	11.4	653	83.7	16.0	0.3	
Tanzania	505	1.21	10.7	2,092	47.1	51.1	1.8	
Vietnam	69	0.97	8.3	546	82.6	17.4	0.0	
**Hookworm**								
Brazil	172	0.62	9.9	420	97.1	2.9	0.0	
Cambodia	160	0.74	9.4	406	98.1	1.3	0.6	
Cameroon	161	0.85	10.3	310	98.1	1.9	0.0	
Ethiopia	100	0.59	11.7	266	100	0.0	0.0	
Tanzania	226	1.35	10.8	854	89.4	7.5	3.1	
Vietnam	80	0.78	8.9	679	93.8	5.0	1.3	

Number of infected subjects (N), sex ratio (number of female subjects/number of male subjects), mean age, mean FEC, and intensity of infection across six mebendazole trials for *Ascaris lumbricoides*, *Trichuris trichiura*, and hookworm infection separately.

**Table 2:** Number of infected subjects and characteristics of the study across seven albendazole trials.

	N	Sex ratio	Mean age (years)	Mean FEC (EPG)	Infection intensity level (%)				
					Low	Moderate			High
***A. lumbricoides***									
Brazil	50	1.00	9.8	9,230	52.0		46.0	2.0	
Cameroon	298	0.77	10.4	12,085	57.4		37.6	5.0	
Ethiopia	151	1.60	11.3	3,443	82.1		17.2	0.7	
Tanzania	279	0.87	9.7	4,280	74.6		25.1	0.4	
Vietnam	148	0.87	9.2	4,742	76.4		22.3	1.4	
***T. trichiura***									
Cameroon	386	0.85	10.7	1,024	79.5		18.4	2.1	
Ethiopia	105	1.10	11.1	420	91.4		8.6	0.0	
Tanzania	396	0.91	9.7	924	67.4		32.6	0.0	
Vietnam	138	1.03	9.4	371	96.4		3.6	0.0	
**Hookworm**									
Brazil	52	0.44	10.3	618	96.2		3.8	0.0	
Cambodia	127	0.63	10.3	586	94.5		3.9	1.6	
Cameroon	140	0.54	11.3	567	95.7		1.4	2.9	
Ethiopia	91	0.90	11.6	326	100		0.0	0.0	
India	95	0.86	11.6	663	92.6		7.4	0.0	
Tanzania	349	0.93	9.8	867	91.4		6.6	2.0	
Vietnam	58	0.71	9.9	205	98.3		1.7	0.0	

Number of infected subjects (N), sex ratio (number of female subjects/number of male subjects), mean age, mean FEC, and intensity of infection across seven albendazole trials for *Ascaris lumbricoides*, *Trichuris trichiura*, and hookworm infection separately.

## Results

**Figure 1:**
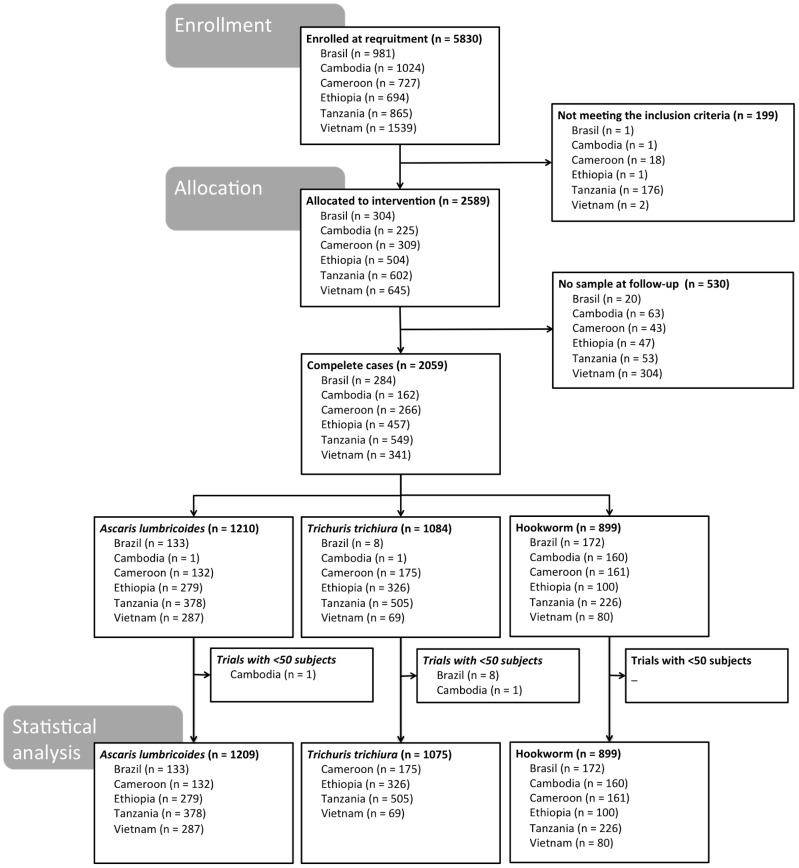
Study participation, occurrence of soil-transmitted helminth infections, and stool sample submission compliance during the mebendazole-intervention. These surveys were conducted in six soil-transmitted helminth-endemic countries (i.e. Brazil, Cambodia, Cameroon, Ethiopia, United Republic of Tanzania, and Vietnam) between December 2011 and April 2012. Subjects who were not able to provide a stool sample for the follow-up, or who were experiencing a severe intercurrent medical condition or had diarrhea at the time of the first sampling, were excluded from the trial.

The numbers of subjects enrolled for the study, allocated to the intervention, receiving treatment, and then providing samples at both pre- and post-intervention surveys are summarized in Figure 1. For infections with each species of STH we also provide the number of subjects who were included in the statistical analysis. Trials in which fewer than 50 subjects carried a specific parasite were not included in the statistical analysis.

### FECR for MEB

**Figure 2:**
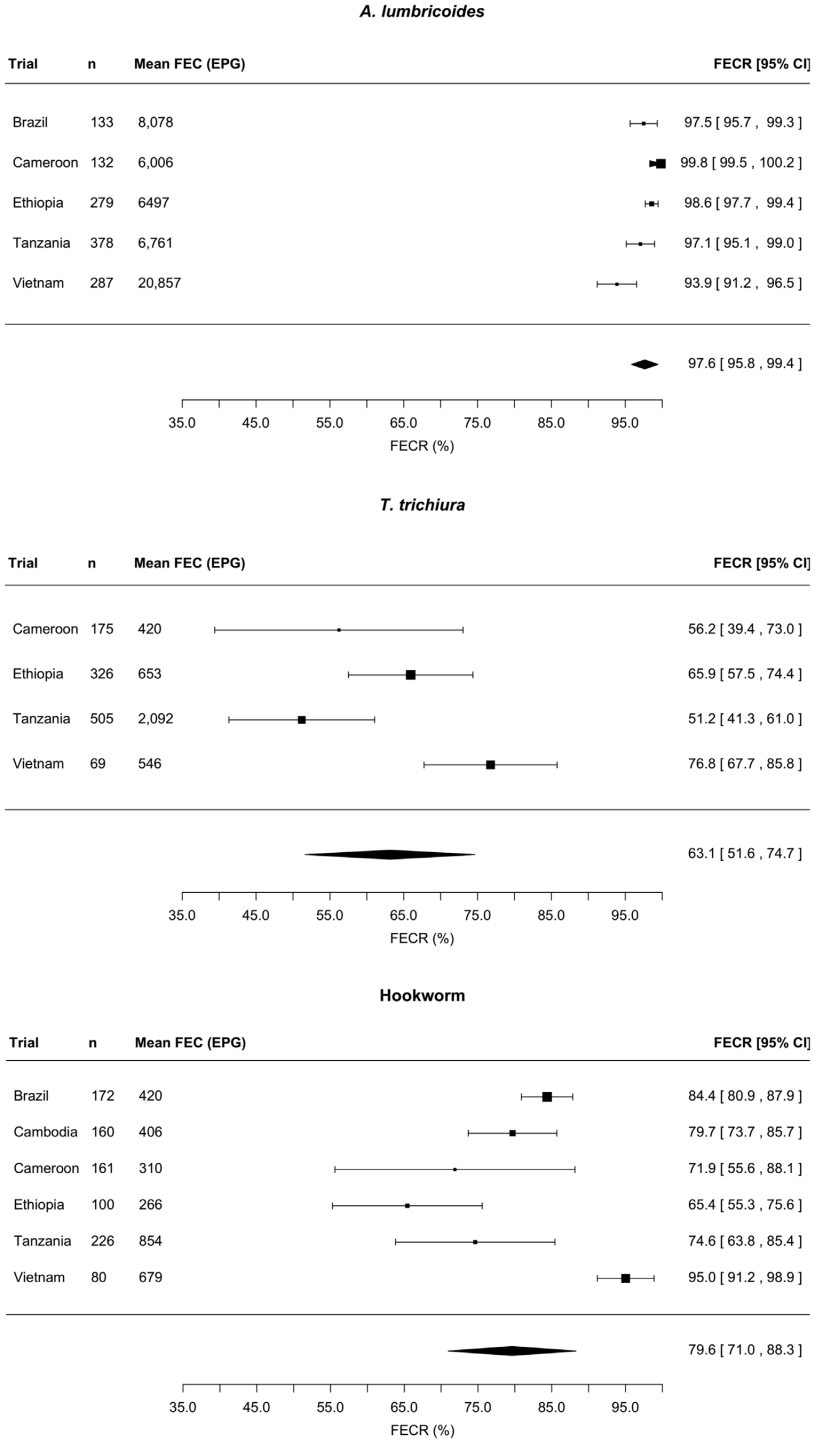
Efficacy of a single dose of mebendazole against soil-transmitted helminths. Three forest plots summarizing the anthelmintic drug efficacy measured by means of fecal egg reduction (FECR) rate of a single oral dose of 500 mg mebendazole against *Ascaris lumbricoides*, *Trichuris trichiura*, and hookworm infections, respectively.

Figure 2 illustrates the outcome of the analyses of FECR of MEB by means of forest plots for *A. lumbricoides*, *T. trichiura*, and hookworm infections. Overall, the estimated FECR rate [95% confidence interval [CI]] of MEB was the highest for *A. lumbricoides* (97.6% [95.8–99.5]), followed by hookworm (79.6% [71.0–88.3]). For *T. trichiura*, the estimated FECR rate was lower, namely 63.1% [51.6–74.6]. An association between the mean FECs at pre-intervention and the FECR rate was observed for *A. lumbricoides* only. For this STH species, the model predicted that the FECR rate would drop by 0.4% as mean FECs at pre-intervention increase by increments of 1,000 EPG (z = -2.97, *p* = 0.003). For the remaining two STHs, there was no significant relationship between FECR rate and mean FEC at pre-intervention (*T. trichiura*: z = 1.46, *p* = 0.144; hookworm: z = 1.00, *p* = 0.316).

### Comparison of FECR between ALB and MEB

**Figure 3:**
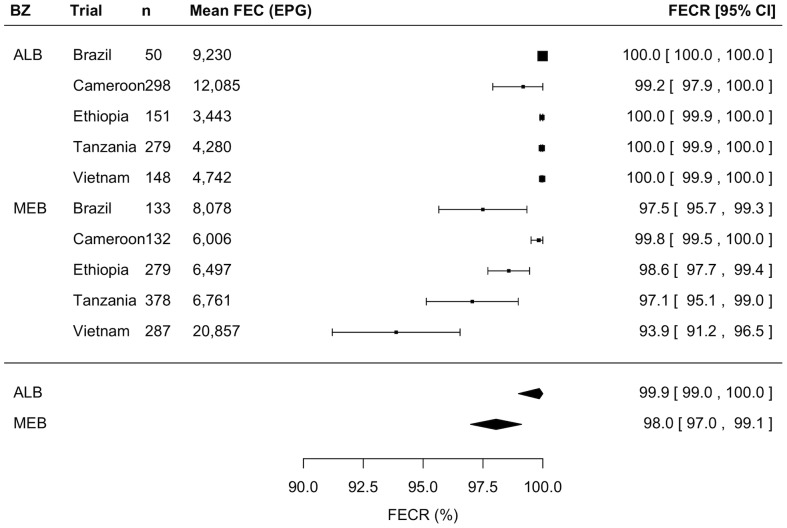
Comparison of efficacy between a single dose of albendazole and mebendazole against *Ascaris lumbricoides* infections. Forest plot comparing the anthelmintic drug efficacy measured by means of fecal egg reduction (FECR) rate of a single oral dose of 400 mg albendazole (ALB) and a single oral dose of 500 mg mebendazole (MEB) against *A. lumbricoides* infection.

**Figure 4:**
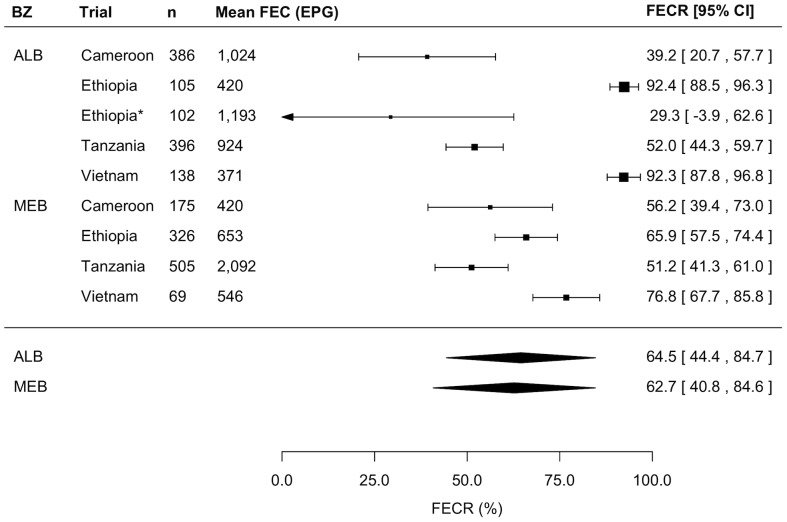
Comparison of efficacy between a single dose of albendazole and mebendazole against *Trichuris trichiura* infection. Forest plot comparing the anthelmintic drug efficacy measured by means of fecal egg reduction (FECR) rate of a single oral dose of 400 mg albendazole (ALB) and a single oral dose of 500 mg mebendazole (MEB) against *T. trichiura* infection.

**Figure 5:**
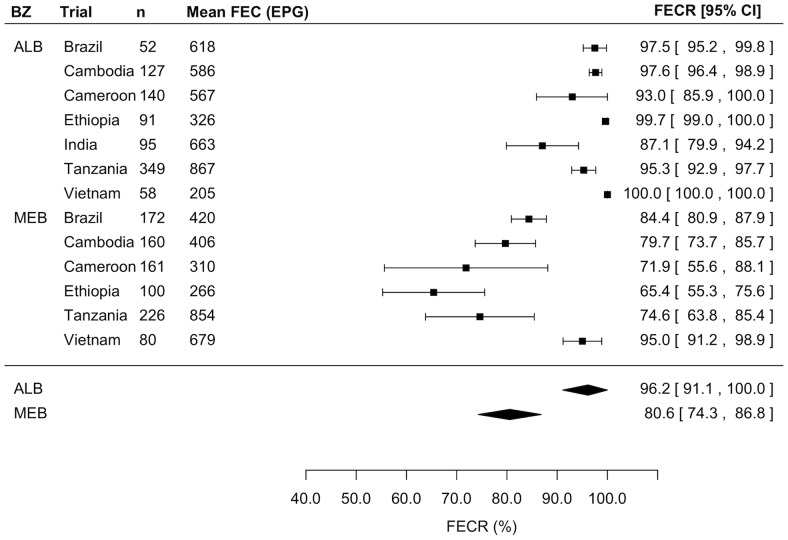
Comparison of efficacy between a single dose of albendazole and mebendazole against hookworm infection. Forest plot comparing the anthelmintic drug efficacy measured by means of fecal egg reduction (FECR) rate of a single oral dose of 400 mg albendazole (ALB) and a single oral dose of 500 mg mebendazole (MEB) against hookworm infections.

Figures 3 to 5 illustrate the outcome of the meta-analyses of the FECR rate against STHs for ALB and MEB by means of forest plots for *A. lumbricoides*, *T. trichiura*, and hookworm infections, respectively. Overall, ALB resulted in statistically higher FECR rates against *A. lumbricoides* (ALB: 99.9% [99.0–100] *vs.* MEB: 98.0% [96.9–99.1], z = −2.5, *p* = 0.012) and hookworm infections (ALB: 96.2% [91.1–100] *vs.* MEB: 80.6% [74.4–86.8], z = 37.4, *p*<0.001). For *T. trichiura* there was no significant difference in FECR rate (ALB: 64.5% [44.4–84.7] *vs.* MEB: 62.7% [40.8–84.6], z = −0.1, *p* = 0.906).

**Figure 6:**
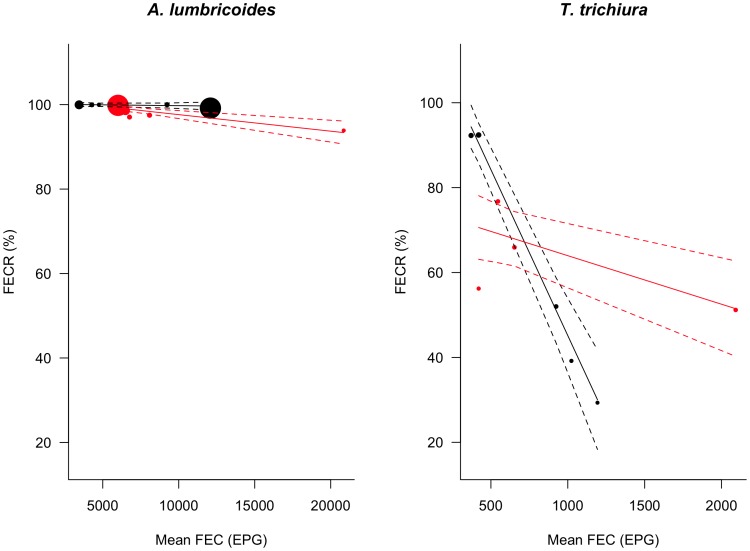
The efficacy albendazole and mebendazole, as a function of infection intensity at the pre-treatment survey. The estimated efficacy measured by means of fecal egg reduction (FECR) rate (straight line) and 95% confidence intervals (dashed line) of a single oral dose of 400 mg ALB (black) and a single dose of 500 mg MEB (red) as a function of infection intensity at the pre-treatment survey (means of FEC) for *Ascaris lumbricoides*, and *Trichuris trichiura.*

Associations between mean FEC at pre-intervention and the FECR rate were observed for both *A. lumbricoides* and *T. trichiura* but in respect of different AE, (Figure 6). For *A. lumbricoides*, the model predicted that the FECR rate after treatment with ALB should remain unchanged across mean FEC at pre-intervention, but that the FECR rate after MEB treatment should fall on average by 0.4% for each 1,000 EPG incremental increase in mean FEC at pre-intervention (interaction term between BZ drug and mean FEC at the pre-intervention survey, z = −2.93, *p* = 0.033). For *T. trichiura*, the model predicted that the FECR rate following treatment with ALB should decrease on average by 7.8% per incremental increase of 100 EPG in mean FEC at pre-intervention ( =  main effect of mean FEC at the pre-intervention, z = −9.1, *p*<0.001). However, there was a significant interaction between BZ drug and mean FEC at pre-intervention (z = 6.9, *p*<0.001), indicating a significant difference between the two BZ drugs in the rate of fall of the FECR rate with increasing pre-intervention FEC. In contrast to the 7.8% fall/100 EPG increment for ALB, for MEB, the model predicted a net fall in FECR rate of only 1.1%/100 EPG increment, which as shown earlier when assessed separately from ALB, did not represent a significant association. For hookworm, the FECR rate of both BZ drugs did not depend significantly on the mean FEC at pre-intervention.

## Discussion

This is the first study that has generated a robust, reliable estimation of the FECR rate following treatment with MEB, and has compared thoroughly the efficacy of MEB with that of ALB, against STH infections. Although we must acknowledge some variation in follow-up period across the trials, both the ALB and MEB trials were standardized at a level unprecedented in the scientific literature [14]. Moreover, most previous studies evaluating drug efficacy of BZ drugs against STHs have generally not summarized their efficacy results by means of the group-based FECR formula, using the arithmetic mean and its corresponding 95% CI, which are now recognized as a suitable, indeed the most informative metric, for the outcome of such trials [14] and are needed to enable a meta-analysis of drug efficacy against STHs [13], .

Overall, the results of this study indicate that a single oral dose of MEB is most efficacious against *A. lumbricoides* infections, followed by hookworm, but that it is relatively inefficacious for infections with *T. trichiura*, thus confirming the earlier efficacy studies reviewed by Bennett and Guyatt [26] and Keiser and Utzinger [13]. The relatively poor efficacy of a single dose treatment with either MEB or ALB in reducing *T. trichiura* FECs is not a novel finding [13], [26], and has resulted in ongoing research on the development and evaluation of new drugs or drug combinations that reduce *T. trichiura* worm burdens more effectively following single dose application, e.g. pyrantel/oxantel [27], mebendazole/ivermectine [28], oxantel, [29], and papaya cysteine proteinases [30]. Based on the overall drug efficacy results for the three STH species, we recommend that monitoring programs of single-dose MEB-dependent PC use a minimum FECR (group-based formula using arithmetic mean) of 95% for *A. lumbricoides*, 70% for hookworm, and 50% for *T. trichiura* as appropriate reference values (as they are below the lower limit of the 95% CI of overall estimates), and that efficacy levels below this should raise concern about the possible emergence of drug resistance.

Compared to a single oral dose of ALB, MEB was significantly less efficacious against hookworm and to a lesser extent against *A. lumbricoides* infections, but equally inefficacious for *T. trichiura* infection. In addition, the efficacies of both ALB and MEB were dependent on the intensity of *A. lumbricoides* and *T. trichiura* infection, decreasing with increasing infection intensity. However, the magnitude of this loss of efficacy as a function of increasing infection intensity differed between the two BZ drugs and the STH species. Between the BZ drugs, the change in drug efficacy was more pronounced for MEB with *A. lumbricoides*, whereas for *T. trichiura* the decrease was more pronounced for ALB. Among STHs, the overall impact of infection intensity on treatment with BZ was pronounced for *T. trichiura* (1.2–7.8% per 100 EPG), but almost negligible for *A. lumbricoides* (0.4% per 1,000 EPG). For hookworm, the efficacy did not depend on the infection intensity. This could be explained by either a true constant efficacy across infection intensities or a low number of moderate and high infection intensities in these trials (see Table 1). The bases of these differences in efficacy between the BZ drugs and their effects on STHs remain unclear, mainly due to the paucity of detailed pharmacokinetic and pharmacodynamics studies in pediatric populations in STH-endemic countries [31].

Currently, recommendations in PC programs are solely based on the overall prevalence of STHs, with these drugs being administered once a year when the STH prevalence is ≥20% and <50%, and twice a year when the prevalence is ≥50% [4]. Although this study indicates that the best choice of BZ drug depends on the relative prevalence and species of STH infections (ALB: hookworm> *T. trichiura*; MEB: *T. trichiura*> hookworm), practical experience with both drugs in the field over several years indicates that both are equally effective in controlling all three STH species irrespective of their initial prevalence and intensity of infection [32], [33]. However, future studies are required to (i) evaluate the difference between BZ drugs in long-term impact (prevalence, infection intensity, and occurrence of single nucleotide polymorphisms in the β-tubulin gene associated with BZ resistance); (ii) determine STH-specific thresholds for infection intensity to justify choice of BZ drugs; and (iii) assess the cost-effectiveness of distributing more than one class of BZ to different regions in a country [12], [34].

In conclusion, our findings suggest that FECR rates exceeding 95% for *A. lumbricoides*, 70% for hookworm, and 50% for *T. trichiura* should be expected in all future surveys, and that any FECR rate below these levels following a single oral dose of MEB (500 mg) should be viewed with concern in light of potential development of drug resistance. In addition, the study highlights the need for detailed pharmacokinetic/pharmacodynamic studies for single-oral dose of BZ drugs in pediatric populations in countries where STHs are endemic to gain additional insights into the observed differences in drug efficacy between ALB and MEB across the various STH species. Finally, additional recommendations advising those running PC programs about which of the BZ drugs to administer in a given setting (i.e., depending on the extent of *T. trichiura* and hookworm infections in a specific location/population) may improve the long-term benefits accruing from PC programs.

## Supporting Information

Study Protocol S1 The study protocol.
(PDF)

Checklist S1 The CONSORT checklist.
(DOC)

## References

[1] Murray CJL, Vos T, Lozano R, Naghavi M, Flaxman AD, et al (2012) Disability-adjusted life years (DALYs) for 291 diseases and injuries in 21 regions, 1990–2010: a systematic analysis for the Global Burden of Disease Study 2010. Lancet 380: 2179–2223. 10.1016/S0140-6736(12)61689-4 23245608

[2] Pullan RL, Smith JL, Jasrasaria R, Brooker SJ (2014) Global numbers of infection and disease burden of soil-transmitted helminth infections in 2010. Parasit Vectors 7: 37. 24447578 10.1186/1756-3305-7-37 PMC3905661

[3] Gabrielli AF, Montresor A, Chitsulo L, Engels D, Savioli L (2011) Preventive chemotherapy in human helminthiasis: theoretical and operational aspects. Trans R Soc Trop Med Hyg 105: 683–693. 22040463 10.1016/j.trstmh.2011.08.013 PMC5576527

[4] WHO (2011) Helminth control in school children: a guide for managers of control programmes. Geneva: World Health Organization.

[5] WHO (2012) Accelerating work to overcome the global impact of neglected tropical diseases: a roadmap for implementation. Geneva: World Health Organization.

[6] Neglected Tropical Disease Partner Website (2012) Uniting to combat neglected tropical diseases. Ending the neglect and reaching 2020 goals. Available at: http://www.unitingtocombatntds.org; accessed 10 April 2014.

[7] WHO (2012) Soil-transmitted helminthiases: eliminating soil-transmitted helminthiases as a public health problem in children: progress report 2001-2010 and strategic plan 2011–2020. Geneva: World Health Organization.

[8] Olliaro P, Seiler J, Kuesel A, Horton J, Clark JN, et al (2011) Potential drug development candidates for human soil-transmitted helminthiases. PLoS Negl Trop Dis 5: e1138. 21695247 10.1371/journal.pntd.0001138 PMC3111745

[9] Vercruysse J, Albonico M, Behnke JM, Kotze AC, Prichard RK, et al (2011) Is anthelmintic resistance a concern for the control of human soil-transmitted helminths? Int J Parasitol Drugs and Drug Resist 1: 14–27. 24533260 10.1016/j.ijpddr.2011.09.002 PMC3913213

[10] Smout MJ, Kotze AC, McCarthy JS, Loukas A (2010) A novel high throughput assay for anthelmintic drug screening and resistance diagnosis by real-time monitoring of parasite motility. PLoS Negl Trop Dis 4: e885. 21103363 10.1371/journal.pntd.0000885 PMC2982823

[11] Kotze AC, Steinmann P, Zhou H, Du ZW, Zhou XN (2011) The effect of egg embryonation on field-use of a hookworm benzimidazole-sensitivity egg hatch assay in Yunnan province, People's Republic of China. PLoS Negl Trop Dis 5: e1203. 21738805 10.1371/journal.pntd.0001203 PMC3125137

[12] Diawara A, Halpenny CM, Churcher TS, Mwandawiro C, Kihara J, et al (2013) Association between response to albendazole treatment and β-tubulin genotype frequencies in soil-transmitted helminths. PLoS Negl Trop Dis 7: e2247. 23738029 10.1371/journal.pntd.0002247 PMC3667785

[13] Keiser J, Utzinger J (2008) Efficacy of current drugs against soil-transmitted helminth infections: systematic review and meta-analysis. JAMA 299: 1937–1948. 18430913 10.1001/jama.299.16.1937

[14] Vercruysse J, Behnke JM, Albonico M, Ame SM, Angebault C, et al (2011) Assessment of the anthelmintic efficacy of albendazole in school children in seven countries where soil-transmitted helminths are endemic. PLoS Negl Trop Dis 5: e948. 21468309 10.1371/journal.pntd.0000948 PMC3066140

[15] Levecke B, Mekonnen Z, Albonico M, Vercruysse J (2011) The impact of baseline FEC on the efficacy of a single-dose albendazole against *Trichuris trichiura* . Trans R Soc Trop Med Hyg 106: 128–130. 22189084 10.1016/j.trstmh.2011.09.007

[16] Levecke B, De Wilde N, Vandenhoute E, Vercruysse J (2009) Field validity and feasibility of four techniques for the detection of Trichuris in simians: a model for monitoring drug efficacy in public health? PLoS Negl Trop Dis 3: e366. 19172171 10.1371/journal.pntd.0000366 PMC2621347

[17] Levecke B, Behnke JM, Ajjampur SS, Albonico M, Ame SM, et al (2011) A comparison of the sensitivity and fecal egg counts of the McMaster egg counting and Kato-Katz thick smear methods for soil-transmitted helminths. PLoS Negl Trop Dis 5: e1201. 21695104 10.1371/journal.pntd.0001201 PMC3114752

[18] Albonico M, Ame SM, Vercruysse J, Levecke B (2012) Comparison of Kato-Katz thick smear and McMaster egg counting method for monitoring drug efficacy against soil-transmitted helminths in school children of Pemba Island, Tanzania. Trans R Soc Trop Med Hyg 106: 199–201. 22261186 10.1016/j.trstmh.2011.11.006

[19] Albonico M, Rinaldi L, Sciascia S, Morgoglione ME, Piemonte M, et al (2013) Comparison of three copromicroscopic methods to assess albendazole efficacy against soil-transmitted helminth infections in school-aged children on Pemba Island. Trans R Soc Top Med Hyg 107: 493–501. 10.1093/trstmh/trt051 23843559

[20] Levecke B, Brooker SJ, Knopp S, Steinmann P, Stothard RJ, et al.. (2014) Effect of sampling and diagnostic effort on the assessment of schistosomiasis and soil-transmitted helminthiasis and drug efficacy: a meta-analysis of six drug efficacy trials and one epidemiological survey. Parasitology (in press). 10.1017/S0031182013002266 24725546

[21] Dobson RJ, Sangster NC, Besier RB, Woodgate RG (2009) Geometric means provide a biased efficacy result when conducting a faecal egg count reduction test (FECRT). Vet Parasitol 161: 162–167. 19135802 10.1016/j.vetpar.2008.12.007

[22] Casella G, Berger BL (2002) Statistical inference. 2^nd^ ed. Duxbury Press. Pacific Grove: Duxbury Thomson Learning.

[23] WHO (1998) Guidelines for the evaluation of soil-transmitted helminthiasis and schistosomiasis at community level. A guide for control programme managers. Geneva: World Health Organization.

[24] Mekonnen Z, Levecke B, Boulet G, Bogers JP, Vercruysse J (2013) Efficacy of different albendazole and mebendazole regimens against high-intensity *Trichuris trichiura* infections in school children, Jimma, Ethiopia. Pathog Glob Health 107: 207–209. 23816513 10.1179/2047773213Y.0000000092 PMC4001473

[25] Viechtbauer W (2010) Coducting meta-analyses in R with the metafor package. J Stat Soft 36: 1–48.

[26] Bennett A, Guyatt H (2000) Reducing intestinal nematode infection: efficacy of albendazole and mebendazole. Parasitol Today 16: 71–74. 10652492 10.1016/s0169-4758(99)01544-6

[27] Albonico M, Bickle Q, Haji HJ, Ramsan M, Khatib KJ, et al (2002) Evaluation of the efficacy of pyrantel-oxantel for the treatment of soil-transmitted nematode infections. Trans R Soc Trop Med Hyg 96: 685–90. 12625151 10.1016/s0035-9203(02)90352-4 PMC5679355

[28] Knopp S, Mohammed KA, Speich B, Hattendorf J, Khamis IS, et al (2010) Albendazole and mebendazole administered alone or in combination with ivermectin against *Trichuris trichiura*: a randomized controlled trial. Clin Infect Dis 51: 1420–1428. 21062129 10.1086/657310

[29] Speich B, Ame SM, Ali SM, Alles R, Huwyler J, et al (2014) Oxantel pamoate-albendazole for *Trichuris trichiura* infection. N Engl J Med 370: 610–20. 24521107 10.1056/NEJMoa1301956

[30] Levecke B, Buttle DJ, Behnke MJ, Duce IR, Vercruysse J (2014) Cysteine proteinases from papaya (*Carica papaya*) in the treatment of experimental *Trichuris suis* infection in pigs: two randomized controlled trials. Parasit Vectors 7: 255. 24886388 10.1186/1756-3305-7-255 PMC4049439

[31] Geary TG, Woo K, McCarthy JS, MacKenzie CD, Horton J, et al (2010) Unresolved issues in anthelmintic pharmacology for helminthiases of humans. Int J Parasitol 40: 1–13. 19932111 10.1016/j.ijpara.2009.11.001

[32] Sinuon M, Tsuyuoka R, Socheat D, Odermatt P, Ohmae H, et al (2006) Control of *Schistosoma mekongi* in Cambodia: results of eight years of control activities in the two endemic provinces. Trans R Soc Trop Med Hyg 101: 34–39. 17028047 10.1016/j.trstmh.2006.04.011 PMC5625998

[33] Flisser A, Valdespino JL, Garcia-Garcia L, Guzman C, Aguirre MT, et al (2008) Using national health weeks to deliver deworming to children: lessons from Mexico. J Epidemiol Community Health 62: 314–317. 18339823 10.1136/jech.2007.066423

[34] Jia T-W, Melville S, Utzinger J, King CH, Zhou X-N (2012) Soil-transmitted helminth reinfection after drug treatment: a systematic review and meta-analysis. PLoS Negl Trop Dis 6: e1621. 22590656 10.1371/journal.pntd.0001621 PMC3348161

