# Supplementary material for: Assessment of Anthelmintic Efficacy of Mebendazole in School Children in Six Countries Where Soil-Transmitted Helminths Are Endemic
Source: PLoS Negl Trop Dis. 2014 Oct 9;8(10):e3204. doi: 10.1371/journal.pntd.0003204 (PMC4191962; doi:10.1371/journal.pntd.0003204)
Supplement: Study Protocol S1 — The study protocol. (PDF) [file pntd.0003204.s001.pdf]

Protocol - Monitoring efficacy of mebendazole for the treatment of Soil Transmitted Helminths

Jozef Vercruysse and Bruno Levecke

Ghent University. WHO Collaborating Centre for the monitoring of anthelminthic drug efficacy for soil-transmitted helminthiasis

Table of Contents

1. Background 1
2. Objectives 3
3. Materials and Methods 3
   3.1. Study sites and population 3
   3.2. Study design 3
   3.3. Parasitological techniques 4
   3.4. Statistical analysis 5
4. Ethical issues 5
5. Starting date 5
6. References 5

1. Background

Soil-Transmitted Helminths

The three major Soil-Transmitted Helminths (STH), *Ascaris lumbricoides* (roundworm), *Trichuris trichiura* (whipworm) and *Necator americanus/Ancylostoma duodenale* (hookworms) are amongst the most widespread parasites worldwide. An estimated 4.5 billion individuals are at risk of STH infection and more than one billion individuals are thought to be infected, of whom 450 million suffer morbidity from their infection, the majority of who are children. An additional 44 million infected pregnant women suffer significant morbidity and mortality due to hookworm-associated anemia. Approximately 135,000 deaths occur per year, mainly due to infections with hookworms or *A. lumbricoides* (Bundy *et al*., 1992; Stoltzfus *et al*., 1996; Crompton *et al*., 2002; WHO, 2005; Bethony *et al*., 2006).

Treatment and monitoring efficacy

The periodic administration of anthelmintics is currently the most widely used method to control these infections. Rather than aiming to achieve eradication, these control programs are focused on reducing infection intensity and transmission potential, primarily to reduce morbidity. The benzimidazole (BZ) drugs, i.e. albendazole (ALB) and mebendazole (MBZ), are the most widely used drugs and a scale-up of these large-scale treatment programs underway in various parts of across Africa, Asia and Latin-America (donation of 400 million tablets of albendazole by GlaxonSmithKline and 200 million tablets of mebendazole by Johnson & Johnson). Due to the scarcity of available anthelmintics, it is imperative that monitoring systems are designed both to assess progress and to detect any changes in therapeutic efficacy that may arise from the selection of worms carrying genes responsible for drug resistance. Although some small-scale studies (De Clercq *et al*., 1997; Albonico *et*

al., 2004) have suggested a reduced efficacy of these components, these studies should be interpreted with some caution. Published studies are confounded by methodological variations including treatment regimens, poor quality of drugs, differing statistical analyses used to calculate therapeutic efficacy, as well as a range of other problems in study design, such as small sample size, diagnostic methods, variation in pre-intervention infection intensities and confounding factors related to geographical locations. A recent multinational trial standardized across different endemic regions using a protocol which was standardized in terms of the treatment (a single-oral 400mg dose of ALB originating from the same batch), the follow up (between 14 and 30 days after) and the detection technique (the McMaster egg counting technique), indicated that ALB is highly efficacious against *A. lumbricoides* and hookworms, but not for *T. trichiura* (Vercruysse *et al*., 2011). MBZ is likely to be more efficacious against *T. trichiura*, yet reliable efficacy data for this BZ are not available.

Animals as reservoir for STH

In addition, it is crucial to identify putative factors contributing to the spread of STH, in order to optimize future control strategies. There is presumptive evidence that animals such as dogs and pigs contribute to the epidemiology of human STH. Dogs have been identified as a reservoir for zoonotic transmission of *Trichuris* (Dunn *et al*., 2002; Traub *et al.,* 2005; Areekel *et al*., 2010) and hookworm infections (Chowdhary and Schad,1972; Traub *et al*., 2008). Despite the fact that patent *Ascaris* infections are rarely found within canines (Shalaby *et al*., 2010), these animals can contribute to the spread of human *Ascaris* by means of mechanical transmitters (Traub *et al.,* 2002). To date, pigs have only been pointed out as a reservoir for human ascariosis (Nejsum *et al.,* 2005; Peng *et al.,* 2007). Their role for the remaining STH parasites is not clear, but positive associations with pig ownership and human trichuriosis and hookworm infections have been observed (Traub *et al*., 2004). Although these epidemiological surveys warrant an integrated control strategy, the importance of animals as a reservoir for human STH remains unclear. This is because the eggs of species of *Ascaris*, *Trichuris* and hookworms are difficult to be differentiated using conventional coproplogical tests. To this end, molecular tools are more appropriate, but these tools have been rarely applied (see next).

Molecular identifications

The relative therapeutic efficacy of both BZ on *N. americanus* and *A. duodenale*is scarce, because the eggs of these species cannot be differentiated using conventional coproplogical tests. For this, coprocultures (Haradi-Mori) need to be used. However, molecular assays would be more appropriate (Verweij *et al*., 2007).

In veterinary medicine, resistance to BZ have been linked to single nucleotide polymorphisms in parasite β-tubulin at codons 167, 198 and/or 200 (Mottier and Prichard, 2008). An increased occurrence of codon 200 in *T. trichiura* was also recently observed in regions where control programs are intensively implemented (Diawara *et al*., 2009). Yet, sample sizes were small, anthelmintic efficacy was not assessed, and treated and non-treated samples were from different locations. Consequently, these frequencies do not allow drawing any conclusions on resistance neither can they be extrapolated to other populations of *T. trichiura*.

2. Objectives

The overall objective is to monitor efficacy of MBZ against STH.

<u>The primary objective is:</u>

(1) to monitor the efficacy a single dose 500 mg of MBZ against STH infections by means of Faecal Egg Count Reduction (FECR) and Cure Rate (CR).

<u>The secondary objectives are:</u>

(1) to assess the occurrence of *Necator americanus* and *Ancylostoma duodenale*

(2) to assess the occurrence of β-tubuline mutations related to resistance before and after drug administration

(3) to evaluate the role of dogs and pigs as reservoir for zoonotic transmission

3. Materials and Methods

3.1. Study sites and population

Study sites

Six out of the seven study sites involved in the 2009 Study (ALB) confirmed their participation, including Brazil (Minas Gerais State), Cambodia (Phnom Phem), Cameroon (Yaoundé), Ethiopia (Jimma), Tanzania (Pemba Island) and Vietnam (Hanoi). India will be replaced by Argentina (Orean region). Each of these study sites has a documented history of STH and drug selection pressure. Moreover, each of them has well equipped diagnostic facilities, skilled personnel.

Study Population

Schoolchildren between 4 and 18 years old are the focus of this study because of two main reasons: School children are normally a major target for regular treatment with anthelminthic, because they are the group that usually has the heaviest worm burdens for *A. lumbricoides* and *T. trichiura*, and are steadily acquiring hookworm infections. In addition, they are in a period of intense physical and intellectual growth (Bundy *et al.,* 1992; Crompton and Nesheim, 2002). Deworming schoolchildren has a considerable benefit on their nutritional status (Stoltzfus *et al*., 1996, Curtale *et al.,* 1995), physical fitness, appetite, growth (Stephenson *et al*., 1993) and intellectual development (Nokes *et al* 1994).

3.2. Study design

Primary objective

Following obtaining informed consent, schoolchildren in the target age range group will be recruited and asked to provide a recent stool sample (an interval of less than 4 hours) that will be processed to determine the FEC for each STH present. For the initial sampling the aim is to enroll at least 250 infected children for at least one of the STH. This sample size was selected based on statistical analysis of study power, using random simulations of correlated over-dispersed FEC data reflecting the variance-covariance structure in a selection of real FEC data sets. This analysis suggested that a sample size of up to 200 individuals ($\alpha = 0.05$, power = 80%) was required to detect a 10 percentage point drop from a null efficacy of ~

80% (mean percentage FEC Δ per individual) over a wide range of infection scenarios. Standard power analyses for proportions also indicated that the detection of a ~10 percentage point drop from a null cure rate required sample sizes up to 200 (the largest samples being required to detect departures from null efficacies of around 50%). Given an anticipated non-compliance rate of 25%, a sample of 250 infected subjects was therefore considered necessary at each study site.
All children providing stool samples will be treated with MBZ single table of 500mg under supervision (chewing + water). The MBZ will be provided (free) by the coordinating group. **Seven up to fourteen days (maximum interval)** after treatment a second faecal sample will be collected from the children to determine again FEC. Subjects who are unable to provide a stool sample at follow-up, or who are experiencing a severe concurrent medical condition or have diarrhea at time of the first sampling, will be excluded from the study.

Secondary objectives
In 5 study sites, faecal samples of 100 infected subjects should be preserved before treatment with MBZ in one tube (1 gram in 10 ml 70% ethanol). Samples of the same children should be also preserved again in one tube (1 gram in 10 ml 70% ethanol) after treatment. Samples have be send to the Laboratory of Parasitology, Ghent University.
The samples, collected **before and after** treatment will be subsequently examined by molecular assays the occurrence *Necator americanus/Ancylostoma duodenale* and the occurrence of β-tubuline mutations related to resistance.
The samples collected **before** treatment will be subsequently examined by molecular assays to assess the role of animals as a reservoir for human STH.

3.3. Parasitological techniques

Determination of FEC of STH
All fecal samples were processed using the McMaster egg counting technique (analytic sensitivity of 50 EPG) for the detection and the enumeration of infections with *A. lumbricoides*, *T. trichiura* and hookworms. All study sites are familiar with the technique and McMaster slides were provided previously.

Molecular assays (Laboratory of Parasitology, Ghent University)
DNA extraction
DNA of STH will be extracted from the samples preserved in ethanol 70% using the Qiagen mini stool kit.

Molecular identification of STH
The presence of the STH species: *Ascaris* (n= 2), *Trichuris* (n = 2) and hookworms (n = 4) will be assessed using different molecular assays (Table 1). For the differentiation of *Trichuris* spp., species-specific polymerase chain reaction (PCR) will be applied. For the differentiation of *Ascaris* and the canine hookworms a PCR-Restriction Fragment Length Polymorphism (PCR-RFLP) will be used. For the human hookworms, a quantitative PCR will be applied.

Table 1. The STH species under examination, their natural host, molecular assay applied

Genus / species	Host	Molecular assay	Reference
Ascaris			
A. lumbricoides	Humans	PCR-RFLP	Zhu *et al*., 1999
A. suum	Pigs		
Trichuris			
T. trichiura	Humans	Species specific PCRs	Areekul *et al*., 2010
T. vulpis	Canidae		
**Hookworms**			
Ancylostoma			
A. duodenale	Humans	qPCR	Verweij *et al*., 2007
A. caninum	Canidae	PCR-RLFP	Palmer *et al*., 2007
A. ceylanicum			
Necator			
N. americanus	Humans	qPCR	Verweij *et al*., 2007

Presence of mutations in β-tubulin related to BZ resistance
This specific objective will be performed in collaboration with McGill University (Canada).

3.4. Statistical analysis
Both CR and FECRT will be considered to monitor to efficacy of MB against STH. The statistical analysis will be assessed as described by Vercruysse *et al*., 2011.

4. Ethical issues
The overall protocol of the study will be reviewed by the Ethics committee of the Faculty of Medicine, Ghent University, Belgium. For each proposed study a separate ethical clearance need to be obtained.

5. Starting date
It is expected to start in end 2011 (if later, please justify)

6. References

- Albonico M, Engels D and Savioli L (2004) Monitoring drug efficacy and early detection of drug resistance in human soil-transmitted nematodes: a pressing public health agenda for helminth control. Int J Parasitol 34, 1205-1210.

- Areekul P, Putaportip C, Pattanawong U, Sitthicharoencai P and Jongwutiwes S (2010) *Trichuris vulpis* and *T. trichiura* among schoolchildren of a rural community in northewestern Thailand: the possible role of dogs in disease transmission. Asian Biomedicine 4, 49-60.

- Bethony J, Brooker S, Albonico M, Geiger SM, Loukas A, et al. (2006) Soil-transmitted helminth infections: ascariasis, trichuriasis, and hookworm. Lancet 367, 1521-1532.

- Bundy DA, Hall A, Medley GF and Savioli L (1992). Evaluating measures to control intestinal parasitic infections. World Health Stat Q45, 168-179.

- Chowdhury, AB and Schad, GA (1972) *Ancylostoma ceylanicum*: a parasite of man in Calcutta and environs. Am J Trop Med Hyg 21, 300-301.

- Crompton DWT and Nesheim MC (2002) Nutritional impact of intestinal helminthasis during the human life cycle. Annu Rev Nutr 22, 35-59.

- Curtale F, Pokhrel RP, Tilden RL and Higashi G (1995) Intestinal helminths and xerophthalmia in Nepal. A case-control study. J Trop Pediatr 41, 334-337.

- De Clercq D, Sacko M, Behnke JM, Gilbert F, Dorny P, et al. (1997) Failure of mebendazole in treatment of human hookworm infections in the Southern Region of Mali. Am J Trop Med Hyg 57, 25- 30.

- Diawara A, Drake LJ, Suswillo RR, Kihara J, Bundy DA et al. (2009) Assays to detect beta-tubulin codon 200 polymorphism in *Trichuris trichiura* and *Ascaris lumbricoides*. PLoS Negl Trop Dis 3(3):e397.

- Dunn JJ, Columbus ST, Aldeen WE, Davis M, and Carroll KC (2002) *Trichuris vulpis* recovered from a patient with chronic diarrhea and five dogs. J Clin Microbiol 40, 2703-2704.

- Montresor A, Ramsan M, Chwaya HM, Ameir H, Foum A, et al. (2001) Extending anthelminthic coverage to non-enrolled school-age children using a simple and low-cost method. Trop Med Int Health 6, 535-537.

- Mottier ML and Prichard RK (2008) Genetic analysis of a relationship between macrocyclic lactone and benzimidazole anthelmintic selection on *Haemonchus contortus*. Pharmacogenet Genomics 18, 128–140.

- Nejsum P, Parker ED, FrydenbergJr J, Roepstorff A, Boes J, et al. (2005) Ascariasis is a zoonosis in denmark. J Clin Microbiol 43, 1142-8.

- Nokes C and Bundy DA (1994) Does helminth infection affect mental processing and educational achievement? Parasitology Today 10, 14-18.

- Palmer CS, Traub RJ, Robertson ID, Hobbs RP, Elliot A, et al. (2007) The veterinary and public health significance of hookworm in dogs and cats in Australia and the status of *A. ceylanicum*. Vet Parasitol 145, 304-413.

- Partnership for Child Development (1999). The cost of large-scale school health programmes which deliver anthelminthics to children in Ghana and Tanzania. Acta Trop 73, 183 - 204.

- Peng W, Yuan K, Hu M and Gasser RB (2007) Recent insights into the epidemiology and genetics of *Ascaris* in China using molecular tools. Parasitology 134, 325-30.

- Shalaby HA, Abdel-Shafy S and Derbala AA (2010) The role of dogs in transmission of *Ascaris lumbricoides* for humans. Parasitol Res 106, 1021–1026.

- Stephenson LS, Latham MC, Adams EJ, Kinoti SN and Pertet A (1993) Physical fitness, growth and appetite of Kenyan school boys with hookworm, *Trichuris trichiura* and *Ascaris lumbricoides* infections are improved four months after a single dose of albendazole. J Nutr 123, 1036-1046.

- Stoltzfus RJ, Albonico M, Chwaya HM, Savioli L, Tielsch J, et al. (1996) Hemoquant determination of hookworm-related blood loss and its role in iron deficiency in African children. Am J Trop Med Hyg 55,399-404.

- Traub RJ, Inpankaew T, Sutthikornchai C, Sukthana Y and Thompson RC (2008) PCR-based coprodiagnostic tools reveal dogs as reservoirs of zoonotic ancylostomiasis caused by *Ancylostoma ceylanicum* in temple communities in Bangkok. Vet Parasitol 155, 67-73.

- Traub RJ, Robertson ID, Irwin PJ, Mencke N and Thompson RC (2005) Canine gastrointestinal parasitic zoonoses in India. Trends Parasitol 21, 42-8.

- Traub RJ, Robertson ID, Irwin PJ, Mencke N and Thompson RC (2002) The role of dogs in transmission of gastrointestinal parasites in a remote tea-growing community in northeastern India. Am J Trop Med Hyg 67, 539-45.

- Traub RJ, Robertson ID, Irwin PJ, Mencke N and Thompson RC (2004) The prevalence, intensities and risk factors associated with geohelminth infection in tea-growing communities of Assam, India. Trop Med Int Health 9, 688-701.

- Vercruysse J, Behnke JM,Albonico M, Ame SM, Angebault C, et al. (2011) Assessment of the anthelmintic efficacy of albendazole in school children in seven countries where soil-transmitted helminths are endemic. PloS Negl Trop Dis 5: e948..

- Verweij JJ, Brienen EA, Ziem J, Yelifari L, Polderman AM, et al. (2007) Simultaneous detection and quantification of *Ancylostoma duodenale*, *Necator americanus*, and *Oesophagostomum bifurcum* in fecal samples using multiplex real-time PCR. Am J Trop Med Hyg 77, 685-90.

- World Health Organization (2005) Deworming for health and development. Report of the third global meeting of the partners for parasitic control. WHO/CDS/CPE/PVC/2005.14, World Health Organization, Geneva.

- Zhu X, Chilton NB, Jacobs DE, Boes Jand Gasser RB (1999) Characterisation of *Ascaris* from human and pig hosts by nuclear ribosomal DNA sequences. Int J Parasitol 29, 358-367.
